# Case Report: Diverse phenotypes of congenital poikiloderma associated with *FAM111B* mutations in codon 628: A case report and literature review

**DOI:** 10.3389/fgene.2022.926451

**Published:** 2022-08-25

**Authors:** Yuhao Wu, Long Wen, Peiru Wang, Xiuli Wang, Guolong Zhang

**Affiliations:** Institute of Photomedicine, Shanghai Skin Disease Hospital, School of Medicine, Tongji University, Shanghai, China

**Keywords:** congenital poikiloderma, FAM111B, mutation, case report, literature review

## Abstract

Congenital poikiloderma is an extremely rare autosomal dominant genetic syndrome, characterized by a combination of early onset poikiloderma, telangiectasia, and epidermal atrophy. *FAM111B* gene with multiple mutations has been identified as a potential causative gene for congenital poikiloderma. In this report, we described a boy with congenital poikiloderma confirmed by clinical manifestations. Next-generation sequencing based on a gene probe panel consisting of 541 genetic loci of genodermatoses, was used to screen mutations of the proband and his parents. Results showed that a missense mutation in the *FAM111B* gene c.1883G>A (rs587777238) was identified in the proband, but absent in his parents, indicating the mutation is *de novo*. In conclusion, a new case of congenital poikiloderma in China was reported, and the hotspot mutations in codon 628 of *FAM111B* gene was reviewed, as well as authenticating the uncertain association between genotypes and phenotypes in this rare disease.

## Introduction

Congenital poikiloderma (hereditary fibrosing poikiloderma) is an extremely rare syndromic form of the autosomal dominant disease. It is characterized by a combination of early onset poikiloderma, telangiectasia, epidermal atrophy, tendon contractures, myopathy, and pulmonary fibrosis (POIKTMP), accompanied with the deficiency of eccrine sweat glands (also called hypohidrosis), sparse scalp hair and absent body hair, including eyebrows and eyelashes (Rayinda et al., 2021). In 2013, family with sequence similarity 111 member B (*FAM111B*) mutations were reported to be responsible for congenital poikiloderma (Mercier et al., 2013). Its mode of inheritance and primary clinical features were first described in two generations of a multiplex South African family. *FAM111B* has also been reported to be associated with inherited exocrine pancreatic dysfunction and prostate cancer (Akamatsu et al., 2012; Seo et al., 2016). In addition, *FAM111B* has been confirmed as a direct target of p53 and identified as an oncogene for lung adenocarcinoma (Sun et al., 2019). However, the underlying pathogenic mechanism concerning *FAM111B* mutations is still unclear.

Herein, we reported a 5-year-old boy with mottled pigmentation, telangiectasia, epidermal atrophy, and a missense mutation (c.1883G>A) of *FAM111B* gene was identified. Furthermore, the mutations in codon 628 of *FAM111B* gene were reviewed and the uncertain association between genotypes and phenotypes in this rare disease was also authenticated.

## Case report

### Ethical approval

The current study conformed to the tenets of the Helsinki declaration and was approved by Ethical Committee of Shanghai skin disease hospital. The proband, his parents and 120 ethnically matched control individuals were informed regarding the purpose of the study and written consent was provided prior to recruitment and sampling.

### Case description

**FIGURE 1:**
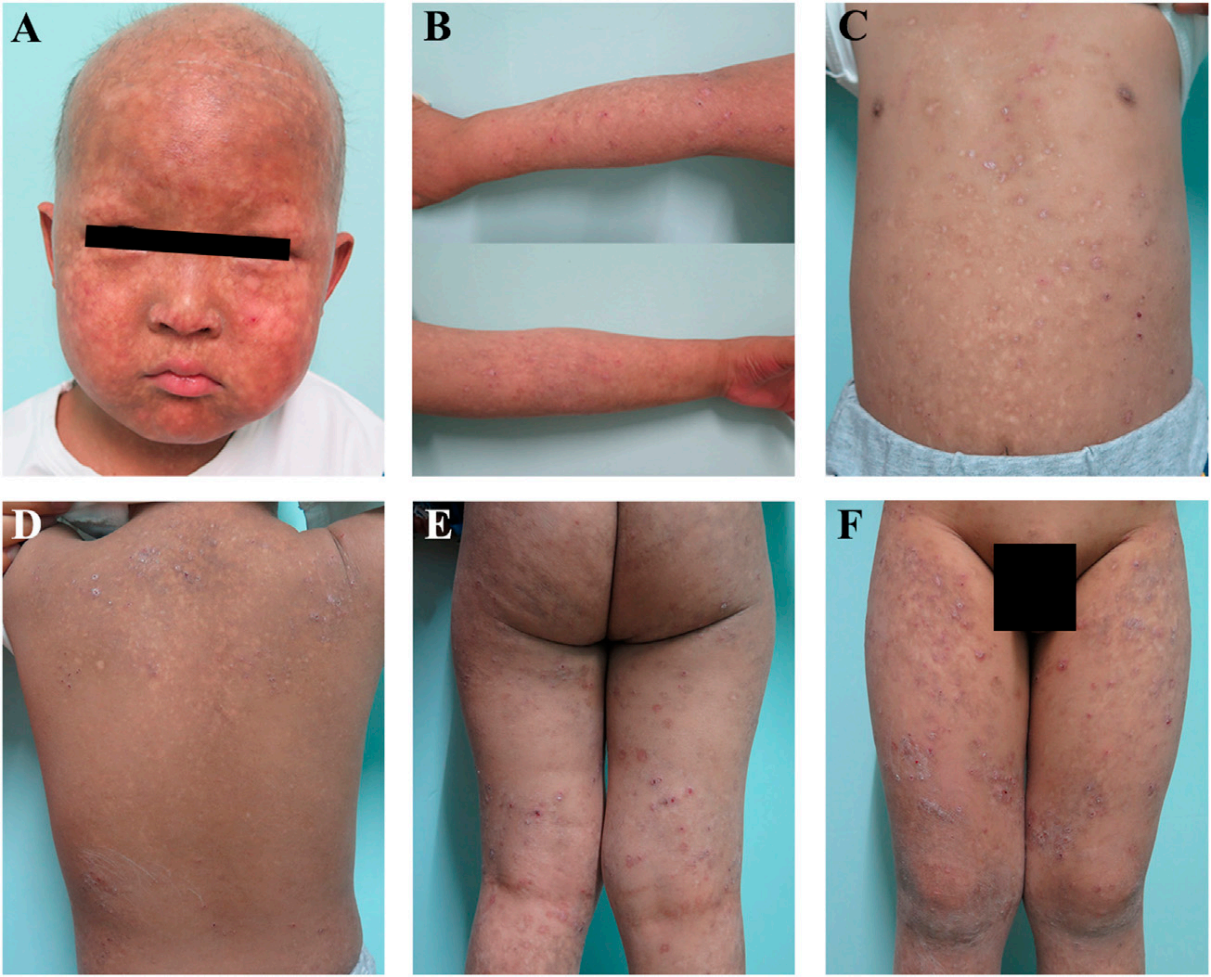
Clinical features of the patient **(A,B)** Congenital poikiloderma, including mottled pigmentation, telangiectasia, epidermal atrophy, sparse scalp hair, as well as absent eyelashes and eyebrows **(C–F)** The patient also had eczematous lesions on the trunk and legs.

A 5-year-old boy was admitted to the Shanghai Skin Disease Hospital outpatient department for developed blisters on the scalp, that were present 1 month after his birth, which gradually spread to the whole body and turned into poikiloderma after 3 months (Figure 1). The lesion was predominantly located on the face and in the other sun-exposed areas, which were typical manifestations for the diagnosis of congenital poikiloderma (Figures 1A,B). He had hypohidrosis and also eczematous lesions on the trunk and legs (Figures 1C–F). No lymphoedema of the upper or lower extremities was observed. Since the onset of the disease, the rash has occurred repeatedly and aggravated in winter. His scalp hair was sparse, with eyelashes and eyebrows absent. His nails and teeth were normal. In addition, from the first year of life, elevated liver transaminase levels were observed on repeat blood samples, including aspartate aminotransferase (316 U/L; normal range, 15–40 U/L), alanine transferase (354 U/L; normal range, 9–50 U/L), γ-glutamyl transferase (334 U/L; normal range, 10–60 U/L), alkaline phosphatase (532 U/L; normal range, 0–500 U/L) and lactate dehydrogenase (362 U/L; normal range, 120–230 U/L). Vasodilation and hyperemia were also observed. The results were consistent with the manifestation in congenital poikiloderma.

### Multi-gene panel sequencing

To investigate the underlying mutation of congenital poikiloderma, next-generation sequencing based on a multi-gene probe panel consisting of 541 genes of monogenic hereditary diseases was used to screen mutations of the proband and his parents. In detail, genomic DNA was extracted from the peripheral blood using the Wizard Genomic DNA purification kit (Promega Corporation). A total of 120 unrelated population-matched control samples were also used to exclude the possibility that these were polymorphisms. Total DNA was isolated from peripheral blood using QIAamp DNA Mini kit (Qiagen, Inc.) according to the manufacturer’s instructions. DNA was concentrated and quality controled using a Qubit 3.0 Fluorometer instrument (Invitrogen; Thermo Fisher Scientific, Inc.) to ensure the concentration was higher than 40 ng/μL. The Illumina Hiseq X Ten sequencing platform (Illumina, Inc.) was used, with an average sequencing depth >200× and Q30 > 90%. To verify the accuracy of the identified mutation, direct Sanger sequencing was performed to confirm whether the variants co-segregated with the disease phenotype in the proband and his parents using an ABI PRISM 3730XL automated sequencer (Applied Biosystems; Thermo Fisher Scientific, Inc.). The sequencing reactions were all performed in forward and reverse directions. The American College of Medical Genetics (ACMG) classification of the variant was performed using the online tool Varsome (https://varsome.com/) (Kopanos et al., 2019).

### Genetic analysis

**FIGURE 2:**
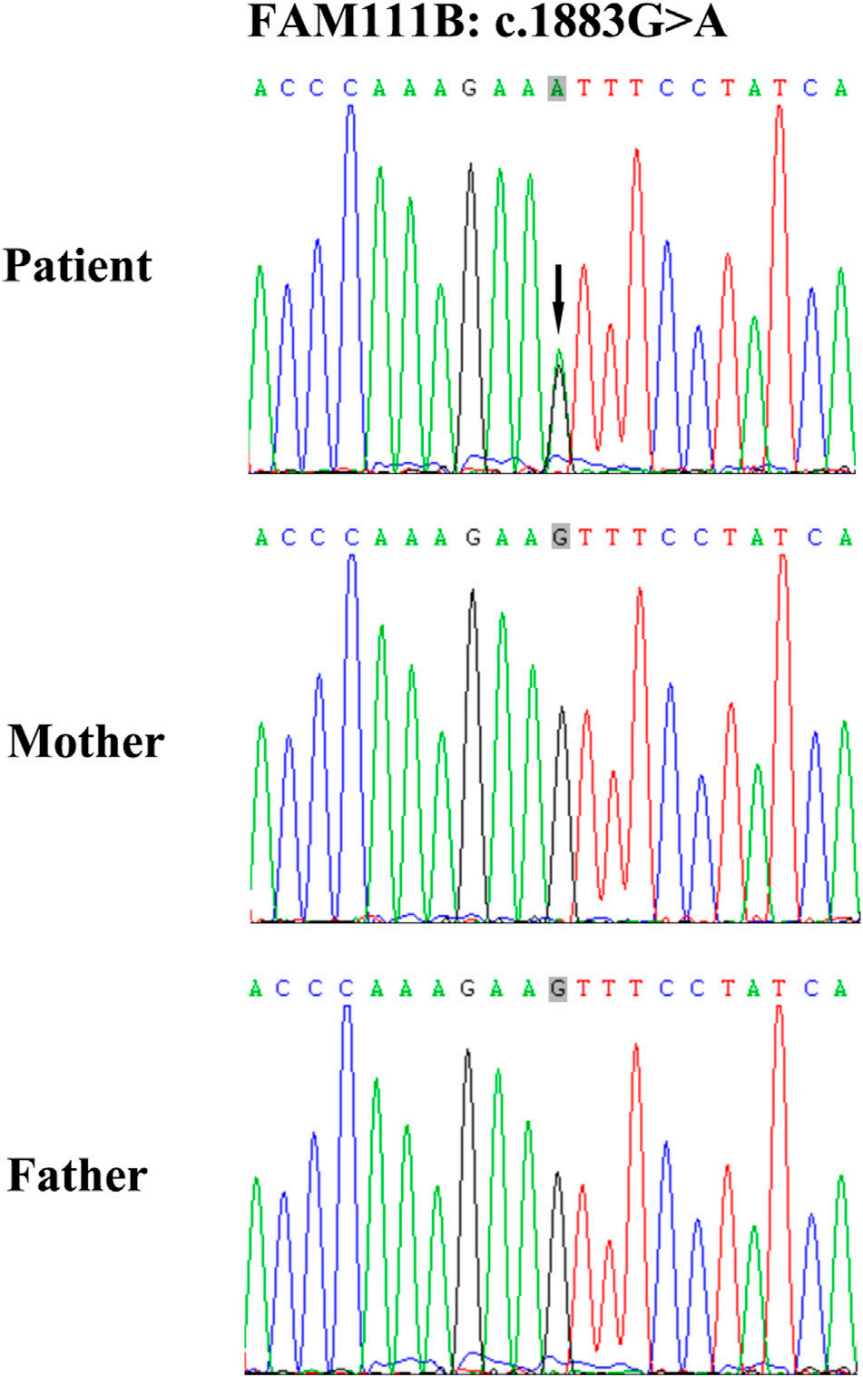
Genomic DNA of the patient was analyzed using a gene probe consisting of 541 genetic loci of Geno dermatoses. Sequences were aligned to GRCh38. The c.1883G>A mutation in exon four exhibited a heterozygous point mutation in the patient, indicated by the black arrow, which was absent in his unaffected parents.

A heterozygous point mutation, c.1883G>A (rs587777238) in *FAM111B* was detected, leading to an amino acid alternation from serine to asparagine (*p*.628S > N) (Figure 2). This mutation was absent from his unaffected parents, which indicates that it is a *de novo* event. According to the ACMG variant classification guideline, this variant was categorized as a pathogenic variant. Moreover, it was not found in any of the healthy controls also showing that it is a novel pathogenic mutation, not a common polymorphism. This mutation causes protein structural and functional changes, which induces the occurrence of this disease.

### Literature review

The following terms were combined in the search strategy [FAM111B (Title/Abstract)] AND [poikiloderma (Title/Abstract)] from PubMed database. Then, the retrieved literatures were analyzed in full text. Mutations in condon 628 of *FAM111B* identified in congenital poikiloderma were summarized.

### Phenotypic heterogeneity for codon 628 in FAM111B

Previous studies suggested that codon 628 of *FAM111B* could be a mutation hotspot. A total of 8 cases with mutations in codon 628 were retrieved from PubMed and results from the present case report were also illustrated for comparison (Supplementary Table S1). Poikiloderma and hypohidrosis were found in every patient carrying *FAM111B* mutations in codon 628. In contrast to the other patients, the proband in our report showed more severe liver damage, while muscle weakness was not found. Patients mostly present with hypotrichosis, but patients in one pedigree reported by Goussot et al. showed no signs of the symptom (Goussot et al., 2017).

## Discussion

Congenital poikiloderma is primarily characterized by early onset poikiloderma, combining with several symptoms, such as telangiectasia and epidermal atrophy, which occurs in neonates and infants. The susceptible gene, *FAM111B*, was identified by Mercier *et al.* (Chen et al., 2019) in 2013, which is the second member of the two-gene “family with sequence similarity 111” gene family. *FAM111B* contains four exons and encodes 734 amino acids, which is likely to contain a trypsin-like cysteine/serine peptidase domain. The identification of mutations in *FAM111B* provided definitive evidence for POIKTMP and distinguishes it from other types of hereditary poikiloderma, such as Rothmund-Thomson syndrome (RTS), hereditary sclerosing poikiloderma of Weary, Kindler syndrome and Clericuzio-type poikiloderma with neutropaenia (Arnold et al., 2010; Küry et al., 2016; Gatinois et al., 2020). In approximately 50% of affected individuals, *FAM111B* pathogenic variant is *de novo* (Mercier et al., 1993), which is the same as the present study.

In the present study, a rare case of congenital poikiloderma with a missense mutation (c.1883G>A) in *FAM111B* was reported. This mutation was within the putative protease domain and predicted to be pathogenic by Varsome database (https://varsome.com/), revealing that the mutation would promote the development of this disease. Recent studies found that disease-associated *FAM111B* mutants forms a complex with Family with sequence similarity 111 member A (*FAM111A*), hyperactivating the intrinsic protease activity of *FAM111A via* a common gain‐of‐function mechanism, which may become the cause of the hereditary fibrosing poikiloderma syndrome (Hoffmann et al., 2020).

Inter-familial phenotypic variability has been observed in congenital poikiloderma, indicating that it may be a multisystem disorder. The same mutation could lead to different phenotypes and an association between genotypes and phenotypes was not established (Mercier et al., 2013; Mercier et al., 2015; Goussot et al., 2017), suggesting that other factors, such as racial factor and environmental variables, might influence the clinical characteristics of this disease. To date, including our case in the present study, a total of 37 patients with this rare disorders have been reported globally (Arowolo et al., 2022a). For patients with congenital poikiloderma, the predominant manifestation is early onset poikiloderma, telangiectasia, and epidermal atrophy. However, patients display a wide spectrum of disease phenotypes. With respect to the genotype-phenotype association, the mutations in the *FAM111B* gene can be classified into two categories according to their positions (Table 1). The codons 621, 625, 627, and 628 are located within the putative protease domain of *FAM111B*, which may be associate with more severe clinical symptoms in skin, muscle and internal organs, and worse prognosis (Arowolo et al., 2022b). The clinical manifestations in affected individuals with mutations located outside the domain, such as codons 416 and 430, may be characterized by sclerosis, lymphoedema, bullous lesions, and pancreatic cancer (Takeichi et al., 2017; Arowolo et al., 2022b). In our reported case, the patient showed no symptoms or had mild symptoms such as tendon contractures and myopathy, which might be a result of the young age. Longer-term clinical follow-up is required.

**TABLE 1:** A comparison of clinical features of different mutation spots of FAM111B.

The FAM111B mutations	Location	Clinical features
Codon 416, 430	Outside the putative protease domain	Poikiloderma, Atopecia, Sclerosis, lymphoedema, bullous lesions, and pancreatic cancer
Codons 621, 625, 627, and 628	Within the putative protease domain	Poikiloderma, Atopecia, telangiectasia, epidermal atrophy, tendon contractures, myopathy, liver damage and pulmonary fibrosis

In conclusion, we reported a new case of congenital poikiloderma with *FAM111B* mutation c.1883G>A in China. Diverse phenotypes of congenital poikiloderma associated with FAM111B mutations in codon 628 were observed. Our results will expand the current knowledge and also verify the incomplete association between genotypes and phenotypes of this extremely rare disorder.

## Data Availability

The datasets for this article are not publicly available due to concerns regarding participant/patient anonymity. Requests to access the datasets should be directed to the corresponding author.

## References

[B1] Akamatsu S. Takata R. Haiman C. A. Takahashi A. Inoue T. Kubo M. (2012). Common variants at 11q12, 10q26 and 3p11.2 are associated with prostate cancer susceptibility in Japanese. Nat. Genet. 44, 426–429. 10.1038/ng.1104 22366784

[B2] Arnold A. W. Itin P. H. Pigors M. Kohlhase J. Bruckner Tuderman L. Has C. (2010). Poikiloderma with neutropenia: A novel C16orf57 mutation and clinical diagnostic criteria. Br. J. Dermatol. 163, 866–869. 10.1111/j.1365-2133.2010.09929.x 20618321

[B3] Arowolo A. Rhoda C. Khumalo N. (2022). Mutations within the putative protease domain of the human FAM111B gene may predict disease severity and poor prognosis: A review of POIKTMP cases. Exp. Dermatol. 31, 648–654. 10.1111/exd.14537 35122327 PMC9344908

[B4] Arowolo A. Rhoda C. Khumalo N. (2022). Mutations within the putative protease domain of the human FAM111B gene may predict disease severity and poor prognosis: A review of POIKTMP cases. Exp. Dermatol. 31, 648–654. 10.1111/exd.14537 35122327 PMC9344908

[B5] Chen F. Zheng L. Li Y. Li H. Yao Z. Li M. (2019). Mutation in FAM111B causes hereditary fibrosing poikiloderma with tendon contracture, myopathy, and pulmonary fibrosis. Acta Derm. Venereol. 99, 695–696. 10.2340/00015555-3186 30938824

[B6] Gatinois V. Desprat R. Pichard L. Becker F. Goldenberg A. Balguerie X. (2020). IPSC reprogramming of fibroblasts from a patient with a Rothmund-Thomson syndrome RTS. Stem Cell Res. 45, 101807. 10.1016/j.scr.2020.101807 32416578

[B7] Goussot R. Prasad M. Stoetzel C. Lenormand C. Dollfus H. Lipsker D. (2017). Expanding phenotype of hereditary fibrosing poikiloderma with tendon contractures, myopathy, and pulmonary fibrosis caused by FAM111B mutations: Report of an additional family raising the question of cancer predisposition and a short review of early-onset poikiloderma. JAAD Case Rep. 3, 143–150. 10.1016/j.jdcr.2017.01.002 28349113 PMC5358901

[B8] Hoffmann S. Pentakota S. Mund A. Haahr P. Coscia F. Gallo M. (2020). FAM111 protease activity undermines cellular fitness and is amplified by gain-of-function mutations in human disease. EMBO Rep. 21, e50662. 10.15252/embr.202050662 32776417 PMC7534640

[B9] Kopanos C. Tsiolkas V. Kouris A. Chapple C. E. Albarca Aguilera M. Meyer R. (2019). VarSome: The human genomic variant search engine. Bioinformatics 35, 1978–1980. 10.1093/bioinformatics/bty897 30376034 PMC6546127

[B10] Küry S. Mercier S. Shaboodien G. Besnard T. Barbarot S. Khumalo N. P. (2016). CUGC for hereditary fibrosing poikiloderma with tendon contractures, myopathy, and pulmonary fibrosis (POIKTMP). Eur. J. Hum. Genet. 24, 779. 10.1038/ejhg.2015.205 PMC4930101 26443268

[B11] Mercier S. Küry S. Salort-Campana E. Magot A. Agbim U. Besnard T. (2015). Expanding the clinical spectrum of hereditary fibrosing poikiloderma with tendon contractures, myopathy and pulmonary fibrosis due to FAM111B mutations. Orphanet J. Rare Dis. 10, 135. 10.1186/s13023-015-0352-4 26471370 PMC4608180

[B12] Mercier S. Küry S. Shaboodien G. Houniet D. T. Khumalo N. P. Bou-Hanna C. (2013). Mutations in FAM111B cause hereditary fibrosing poikiloderma with tendon contracture, myopathy, and pulmonary fibrosis. Am. J. Hum. Genet. 93, 1100–1107. 10.1016/j.ajhg.2013.10.013 24268661 PMC3853004

[B13] Mercier S. Küry S. Barbarot S. (1993). “Hereditary fibrosing poikiloderma with tendon contractures, myopathy, and pulmonary fibrosis,” in GeneReviews(®). Editors Adam M. P. Mirzaa G. M. Pagon R. A. Wallace S. E. Bean L. J. H. Gripp K. W. (Seattle (WA): University of Washington, Seattle. GeneReviews is a registered trademark of the University of Washington, Seattle. All rights reserved.). Copyright © 1993–2022. 27748098

[B14] Rayinda T. Steensel M. Danarti R. (2021). Inherited skin disorders presenting with poikiloderma. Int. J. Dermatol. 60, 1343–1353. 10.1111/ijd.15498 33739439

[B15] Seo A. Walsh T. Lee M. K. Ho P. A. Hsu E. K. Sidbury R. (2016). FAM111B mutation is associated with inherited exocrine pancreatic dysfunction. Pancreas 45, 858–862. 10.1097/MPA.0000000000000529 26495788 PMC4841754

[B16] Sun H. Liu K. Huang J. Sun Q. Shao C. Luo J. (2019). FAM111B, a direct target of p53, promotes the malignant process of lung adenocarcinoma. Onco. Targets. Ther. 12, 2829–2842. 10.2147/OTT.S190934 31114230 PMC6489872

[B17] Takeichi T. Nanda A. Yang H. S. Hsu C. K. Lee J. Y. Y. Al-Ajmi H. (2017). Syndromic inherited poikiloderma due to a de novo mutation in FAM111B. Br. J. Dermatol. 176, 534–536. 10.1111/bjd.14845 27406236

