# Supplementary material for: Case Report: Diverse phenotypes of congenital poikiloderma associated with FAM111B mutations in codon 628: A case report and literature review
Source: Front Genet. 2022 Aug 25;13:926451. doi: 10.3389/fgene.2022.926451 (PMC9452834; doi:10.3389/fgene.2022.926451)
Supplement: Supplementary file 1 [file Table1.docx]

Supplementary Table 1 - Clinical and molecular data of affected individuals with *FAM111B* mutations in codon 628.

| Characteristics | | Our patient | Patient 1 | Patient 2 | Patient 3 | Patient 4 | Family 1 | | | |  |
| --- | --- | --- | --- | --- | --- | --- | --- | --- | --- | --- | --- |
|  |  |  |  |  |  |  | Patient 5 | Patient 6 | Patient 7 | Patient 8 |  |
|  | |  | Mercier S (2013) | Mercier S (2015) | Mercier S (2015) | Mercier S (2015) | Goussot R (2017) | | | |  |
| General | Sex | male | female | female | female | male | male | male | female | female |  |
|  | Origin | China | France/Morocco | France | Ireland | Dominican Republic | n/a | n/a | n/a | n/a |  |
|  | Age at last examination | 5 years | 9 years | 4 years | 5 years | 23 years | 64 years | 30 years | 27 years | 6 months |  |
|  | Delayed puberty | - | n/a | n/a | n/a | - | n/a | n/a | n/a | n/a |  |
| Skin | Congenital poikiloderma | + | + | + | + | + | + | + | + | + |  |
|  | - Face | + | + | + | + | + | + | + | +(mainly) | n/a |  |
|  | - Exposed area | + | + | + | - | + | + | n/a | n/a | n/a |  |
|  | - Upper and/or lower limbs | + | + | + | + | + | + | n/a | + | n/a |  |
|  | Eczematiform lesions | + | + | + | + | - | n/a | n/a | + | n/a |  |
|  | Psoriasiform lesions | - | + | - | - | - | n/a | n/a | n/a | n/a |  |
|  | Bullous lesions | +(1 month) | - | - | + | - | n/a | n/a | n/a | n/a |  |
|  | Sclerosis of the digits | - | - | - | - | - | n/a | n/a | n/a | n/a |  |
|  | Lymphoedema of extremities | - | + | + | + | + | + | + | + | n/a |  |
|  | Hypohidrosis | + | + | + | + | + | + | + | + | + |  |
| Hair | Hypotrichosis/Alopecia | + | + | + | + | + | n/a | - | n/a | n/a |  |
|  | - Scalp hair | + | + | + | + | + | n/a | - | n/a | n/a |  |
|  | - Eyebrows | + | + | + | + | + | n/a | n/a | n/a | n/a |  |
|  | - Eyelashes | + | + | + | + | + | n/a | n/a | n/a | n/a |  |
| Nails | Dysplasia | - | - | - | + | + | - | - | n/a | n/a |  |
| Muscle | Muscle weakness(age of onset) | - | +(14 months) | - | +(infancy) | +(2 years) | n/a | n/a | n/a | n/a |  |
|  | - Proximal lower limbs | - | + | - | + | + | n/a | n/a | n/a | n/a |  |
|  | - Distal lower limbs | - | + | - | - | + | n/a | n/a | n/a | n/a |  |
|  | - Proximal upper limbs | - | + | - | - | + | n/a | n/a | n/a | n/a |  |
|  | - Distal upper limbs | - | + | - | - | + | n/a | n/a | n/a | n/a |  |
|  | - Neck extensors | - | + | - | - | + | n/a | n/a | n/a | n/a |  |
|  | Amyotrophy | - | + | - | - | - | n/a | n/a | + (thenar and hypothenar eminences) | + |  |
|  | Abolition of lower limb tendon reflex | - | + | - | n/a | + | n/a | n/a | n/a | n/a |  |
|  | Tendon lengthening | - | - | - | - | - | n/a | n/a | n/a | n/a |  |
| Joints | Lower limbs contractures (Age at onset) | - | Triceps surae | Triceps surae | Triceps surae | - | + | n/a | n/a | n/a |  |
|  |  |  | (2 years) | (3 years) | (4 years) |  |  |  |  |  |  |
|  |  |  | Hamstring (7 years) |  |  |  |  |  |  |  |  |
|  | Upper-limb contractures | - | + | - | - | + | + | n/a | n/a | n/a |  |
| Lung | Restrictive syndrome | - | + | - | + | + | - | n/a | - | n/a |  |
| Oral sphere | Dysphagia/Velopharyngeal insufficiency | - | + | - | - | + | n/a | n/a | n/a | n/a |  |
| Liver | Hepatomegaly | - | - | - | - | - | n/a | n/a | n/a | n/a |  |
| Pancreas | Steatorrhea/Exocrine insufficiency/Others | - | - | + | + | + | pancreatic cancer | - | - | - |  |
| Eye | Cataract | - | - | - | - | - | - | n/a | n/a | n/a |  |
| Blood Test | Liver function | AST: 316 IU/L (<40), | n/a | SGOT: 63 IU/L (<53); | SGOT:210 IU/L (<40); | SGOT: 100 IU/L (<38); | n/a | n/a | liver transaminases↑ | n/a |  |
|  |  | ALT: 354 IU/L (<50), |  | SGPT: 56 IU/L (<36); | SGPT: 151 IU/L (<35); | SGPT: 132 IU/L (<41); |  |  |  |  |  |
|  |  | GGT: 334 IU/L (<60) |  | ALP: 308 IU/L (<335); | ALP: 772 IU/L (<315); | ALP:129 IU/L (<129); |  |  |  |  |  |
|  |  | ALP: 532 IU/L (<500) |  | GGT: 53 IU/L (<26) | Bili: 33 mmol/l (<14) | GGT:106 IU/L (<58) |  |  |  |  |  |
|  |  | LDH: 362 U/L (<230) |  |  |  |  |  |  |  |  |  |
|  | Others | Mb: 452.88ng/ml (<70.00) | SCK: 340 IU/I | SCK: 370 IU/I | - | SCK: 372 IU/I | n/a | n/a | n/a | n/a |  |
| Mutations | *FAM111B* | c.1883G>A | c.1883G>A | c.1883G> A | c.1883G> A | c.1883G> A | c.1884T>A | c.1884T>A | c.1884T>A | c.1884T>A |  |
|  |  | (p.Ser628Asn) | (p.Ser628Asn) | (p.Ser628Asn) | (p.Ser628Asn) | (p.Ser628Asn) | (p.Ser628Arg) | (p.Ser628Arg) | (p.Ser628Arg) | (p.Ser628Arg) |  |
|  | Other genes | *AAGAB* (c.833C>T) | *SMN1* (no mutation) | - | - | *CLCN1*- c.2509-3C>T (intronic between exons 22 and 23); | n/a | n/a | n/a | n/a |  |
|  |  |  |  |  |  |  |  |  |  |  |  |
|  |  |  |  |  |  | c.2926 C > T |  |  |  |  |  |
|  |  |  |  |  |  | (p.976 R > X, nonsense mutation) |  |  |  |  |  |
|  |  |  |  |  |  |  |  |  |  |  |  |
| Reference | |  | (2) | (3) | (3) | (3) | (8) | (8) | (8) | (8) |  |

The DNA Sequences of the present study were aligned to the human genom assembly of GRCh38, while other studies did not indicate the used human genome assembly version explicitly. The following abbreviations are used: AST, Aspartate aminotransferase; ALT, Alanine aminotransferase; ALP, Alkaline phosphatase; GGT, γ-glutamyl transpeptidase; Mb, Myoglobin; LDH, Lactate dehydrogenase; SGOT, serum glutamate oxaloacetic transaminase; SGPT, serum glutamate pyruvate transaminase; SCK, serum creatine kinase; -, negative; +, positive; n/a, not applicable.
